# Misophonia: Analysis of the neuroanatomic patterns at the basis of psychiatric symptoms and changes of the orthosympathetic/ parasympathetic balance

**DOI:** 10.3389/fnins.2022.827998

**Published:** 2022-08-11

**Authors:** Elena Grossini, Alessandro Stecco, Carla Gramaglia, Daniel De Zanet, Roberto Cantello, Benedetta Gori, Davide Negroni, Danila Azzolina, Daniela Ferrante, Alessandro Feggi, Alessandro Carriero, Patrizia Zeppegno

**Affiliations:** ^1^Laboratory of Physiology, Department of Translational Medicine, University of Eastern Piedmont, Novara, Italy; ^2^Radiology Unit, Department of Translational Medicine, University of Eastern Piedmont, Novara, Italy; ^3^Psychiatry Unit, Department of Translational Medicine, University of Eastern Piedmont, Novara, Italy; ^4^Neurology Unit, Department of Translational Medicine, University of Eastern Piedmont, Novara, Italy; ^5^Statistic Unit, Department of Translational Medicine, University of Eastern Piedmont, Novara, Italy

**Keywords:** anatomic pathways, autonomic system, functional magnetic resonance, psychometric assessment, trigger sounds

## Abstract

**Background/Aim:**

Misophonia is a disorder characterized by reduced tolerance to specific sounds or stimuli known as “triggers,” which tend to evoke negative emotional, physiological, and behavioral responses. In this study, we aimed to better characterize participants with misophonia through the evaluation of the response of the autonomic nervous system to “trigger sounds,” a psychometric assessment, and the analysis of the neurological pathways.

**Materials and methods:**

Participants included 11 adults presenting with misophonic disturbance and 44 sex-matched healthy controls (HCs). Following recently proposed diagnostic criteria, the participants listened to six “trigger sounds” and a “general annoyance” sound (baby crying) during a series of physiological tests. The effects were examined through functional magnetic resonance imaging (fMRI), the analysis of heart rate variability (HRV), and of galvanic skin conductance (GSC). The fMRI was performed on a 3T Scanner. The HRV was obtained through the analysis of electrocardiogram, whereas the GSC was examined through the positioning of silver-chloride electrodes on fingers. Furthermore, the psychometric assessment included questionnaires focused on misophonia, psychopathology, resilience, anger, and motivation.

**Results:**

Participants with misophonia showed patterns of increased sympathetic activation in response to trigger sounds and a general annoyance sound, the low frequency (LF) component of HRV, the sympathetic index, and the number of significant GSC over the threshold, where the amplitude/phasic response of GSC was higher. The fMRI analysis provided evidence for the activation of the temporal cortex, the limbic area, the ventromedial prefrontal/premotor/cingulate cortex, and the cerebellum in participants with misophonia. In addition, the psychometric assessment seemed to differentiate misophonia as a construct independent from general psychopathology.

**Conclusion:**

These results suggest the activation of a specific auditory-insula-limbic pathway at the basis of the sympathetic activation observed in participants with misophonia in response to “trigger and general annoyance sounds.” Further studies should disentangle the complex issue of whether misophonia represents a new clinical disorder or a non-pathological condition. These results could help to build diagnostic tests to recognize and better classify this disorder. The relevance of this question goes beyond purely theoretical issues, as in the first case, participants with misophonia should receive a diagnosis and a targeted treatment, while in the second case, they should not.

## Introduction

The recent consensus work of experts has led to the definition of misophonia as a reduced tolerance to specific sounds or stimuli related to them (Ferrer-Torres and Giménez-Llort, 2022). These “trigger” stimuli can generate strong negative emotional, physiological, and behavioral reactions that are not commonly observed by the majority of people (Sweedo et al., 2022). For individuals with misophonia, it is difficult to distract themselves from trigger sounds, and they may experience a range of unpleasant consequences, such as suffering, distress, overall functioning impairment, and mental health problems. In individuals suffering from misophonia, symptoms should not be better explained by any co-occurring disorder, including psychiatric conditions or disorders, such as anxiety disorders, mood disorders, personality disorders, obsessive, compulsive related disorders, post-traumatic stress disorder, autism spectrum disorder, and attention deficit hyperactivity disorder.

Past studies showed that subjects with misophonia can exhibit a wide range of “triggers” (Daniels et al., 2020; Jager et al., 2020a), which can be different from one subject to another. Although each person can have his/her own “trigger” stimuli, there are some that, more than others, can better serve as “misophonic triggers.” In particular, sounds associated with oral functions like chewing, eating, and swallowing are the most often reported as misophonic triggers. Also, sounds produced by other people, such as pen clicking, keyboard typing, finger or foot tapping, and shuffling footsteps often serve as “triggers.”

In response to those “trigger” stimuli, individuals with misophonia may experience anger, irritation, anxiety, and aggressive impulses, as well as symptoms related to autonomic nervous system activation, such as motor tics and increased heart rate (Edelstein et al., 2013; Webber et al., 2014; Palumbo et al., 2018). In addition, patients often develop coping mechanisms, such as avoiding social situations in which the trigger stimuli might occur (Schröder et al., 2013; Taylor et al., 2014; Potgieter et al., 2019). To date, it has been clarified that misophonia is an affective auditory processing disorder (Edelstein et al., 2013; Erfanian et al., 2017; Kumar et al., 2017) associated with an increased number of connections, or strength of connections, between the limbic and sympathetic nervous systems, which can cause abnormal processing of sound stimuli. In participants with misophonia, increased functional connectivity within brain regions like the anterior insular cortex (AIC), the anterior cingulate cortex, the ventromedial prefrontal cortex, the posteromedial cortex (posterior cingulate and retrosplenial cortex), the hippocampus, and the amygdala (Kumar et al., 2017) could reflect abnormal salience attribution to misophonic stimuli (Schröder et al., 2019; Seeley, 2019). In particular, it is noteworthy that the ventromedial prefrontal cortex and posteromedial cortex are core parts of the default mode network (DMN) (Raichle et al., 2001), which are connected to AIC and are activated when subjects are engaged in internally directed thoughts and retrieval of memories (Huijbers et al., 2011).

Repeated exposure to the same cues will amplify the salience network activity. The causal mechanism of this phenomenon reflects a conditioned response in which the initially neutral stimulus is increasingly associated with intensified aversive emotions and augmented by increased vigilance (Schröder et al., 2019). Among those areas, the anterior cingulate cortex and insular activity have been linked to increased cardiovascular arousal (Critchley, 2005). This statement was confirmed by the study of Kumar et al. (2017), which showed that the augmented heart rate (HR) and galvanic skin response by “trigger sounds” were mediated by the activity of AIC in participants with misophonia.

Therefore, the nervous structures mentioned above could represent the anatomical basis of the symptoms related to the activation of the autonomic nervous system that characterizes misophonia and could explain the visceral responses associated with emotions (Schwartz et al., 1981).

Recently, increased connectivity between both the auditory and the visual cortex, and between those brain regions and the ventral premotor cortex, has been reported in response to trigger sounds in subjects with misophonia (Kumar et al., 2021). This is of interest as the ventral premotor cortex is responsible for orofacial movement (Grabski et al., 2012). These findings support a model of misophonia based on “hyper-mirroring” according to which sounds would be the “medium” through which actions of other people are excessively mirrored.

Although knowledge about misophonia has increased, a complete analysis of the neuropsychiatric features, of the autonomic nervous system activation and of the neuronal pathways in participants with misophonia (Sweedo et al., 2022) in response to “trigger” sounds, is still lacking. In particular, previous studies have focused either on only one of the aspects mentioned above or have only partially investigated the activation of the autonomic nervous system by recording a few parameters related to the orthosympathetic/parasympathetic nervous system. Furthermore, the sample size calculation was not performed.

In the present study, we aimed to better characterize misophonia through the integrated evaluation of the physiologic, psychiatric, and neurological correlates in response to a trigger sound protocol in participants with misophonia. In particular, our primary endpoint was the analysis of the orthosympathetic/parasympathetic balance during the application of six “trigger sounds” and one “general annoyance” sound. To reach our primary endpoint, the sample size was calculated on the basis of previously reported tests related to the activation of the orthosympathetic nervous system in participants with misophonia. The data obtained in the present study were correlated with the psychometric assessment and the anatomic pathways activated during the trigger sounds protocol, which was investigated through functional magnetic resonance imaging (fMRI).

## Materials and methods

We performed a double-blind case-control study by comparing subjects complaining of misophonic disturbances (participants with misophonia, *N* = 11) to gender-matched healthy controls (HCs) (*N* = 44) on the following variables: the fMRI, the analysis of heart rate variability (HRV), galvanic skin conductance (GSC), and psychiatric assessment. The study was approved by the local Ethical Committee, Azienda Ospedaliero-Universitaria Maggiore della Carità, Novara (CE 81/18). Participant recruitment took place from April 2018 to January 2020; subjects (or their legal representatives) were asked to sign a written informed consent and were treated according to *Good Clinical Practice* principles (Declaration of Helsinki: 2013).

The participants with misophonia were recruited from the community through the online misophonia support group (Misofonia Italia in Facebook). The HCs were recruited through local advertisements. The recruitment of the participants was carried out through an interview conducted according to the model proposed by Sanchez and da Silva (Schröder et al., 2013; Sanchez and da Silva, 2018). The selection was executed by an experienced psychiatrist.

Other potentially comorbid medical conditions were investigated as well.

Inclusion criteria were as follows: age ≥14 years, presence of at least one misophonic symptom, and written informed consent by each subject or parents.

Exclusion criteria were as follows: diagnosis of cardiac or neurologic disease, diabetes, intellectual disability/dementia/cognitive impairment, autoimmune/inflammatory diseases, pregnancy, use of medications, such as α or β blockers, diuretics, calcium channels blockers, smoking habits (>1 cigarette day), use of alcohol/psychoactive substances, unwillingness to participate and/or to sign the written informed consent. Participants were recruited based on the evidence of near-normal hearing as documented from previous visits relating to occupational medicine.

The participants with misophonia and HCs were instructed to abstain from caffeinated beverages for 24 h and from moderate or strenuous physical activity for 48 h before the analyses.

### Trigger sounds protocol

Before beginning the study, the subjects were given a detailed explanation of the experimental procedures. They were instructed to fast for at least 3 h before the beginning of the experiment and all the evaluations were conducted from 9 a.m. to 1.30 p.m. All the participants underwent a rest period lasting 15 min before the start of the procedures (psychometric assessment, HRV, GSC, and fMRI analysis), in a quiet room with a controlled temperature between 26 and 27°C. We have chosen this temperature because it is the one that is reported to be the most comfortable for patients subjected to HRV analysis (Liu et al., 2008). In addition, the results of the GSC measurement are not affected by the selected temperature range (Doberenz et al., 2011). All evaluations were performed in a blinded condition.

In each subject, six different stimulations with sounds that have been widely reported as “triggers” (Edelstein et al., 2013; Kumar et al., 2017; Sweedo et al., 2022) were administered in the following sequence to avoid any bias when interpreting the statistical results: crunchy sound, nails tapping, chewing sound, fast breathing, typing, and click pen sound. Baby crying was used as the “general annoyance” sound, too. The audio clips were selected from YouTube and were composed by means of Audacity software (free and open-source digital audio editor and recording application software licensed under GPL-2.0) which allowed us to remove the background noise and adjust the volume to 70 dB HL. In particular, the sounds were adjusted by selecting the individual parts to get a normalization, in order to have a uniform amplitude. This was done by normalizing the peak width between 0.1 and 0.5 dB HL. As for the measurement of the output volume in headphones, Audacity has its own dB measurement system. The soundtracks were administered to the participants with misophonia and HCs by Beats Solo2 Headphones, with a sensitivity of 115 dB/mW, and an impedance of 45 ohms.

Moreover, each sound did not exceed the 70 dB HL limit, so as to make the experimental conditions as similar as possible to the real context.

**FIGURE 1:**
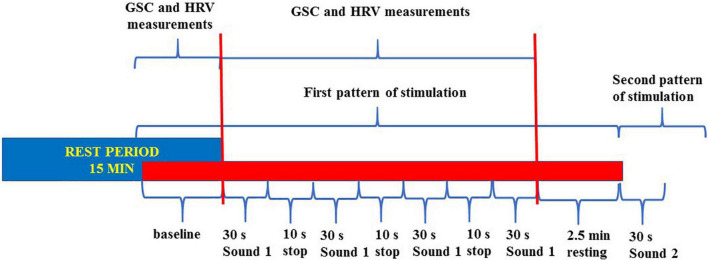
Flowchart about the trigger sounds protocol. The entire sounds stimulation protocol was made of seven patterns of stimulation (six “trigger sounds” and one “annoyance sound”). All patterns were included in a single track. We measured the baseline GSC and HRV variables during each period of stimulation for comparison with the same values at baseline. GSC, galvanic skin conductance; HRV, heart rate variability.

The pattern of stimulation for each sound was as follows: 5 s silence, 30 s sound, 10 s silence, 30 s sound, 10 s silence, 30 s sound, 10 s silence, 30 s sound, and 5 s silence (total period of stimulation for each sound: 2 min and 40 s; five periods of silence and four sound applications; Figure 1).

This pattern of stimulation was preceded and followed by 2 min and 20 s, to allow the return to the baseline before the following stimulation, respectively.

The trigger sounds protocol was similar, in terms of duration and repetition of each sound/silence period, for the psychiatric assessment and the analysis of HRV and GSC. The only difference was during fMRI, where the silence period was 30 s instead of 10 s; this was due to the technical specifications of the fMRI equipment. Thus, the overall time of stimulation was about 42 min.

### Psychometric assessment

Each participant was assessed with a protocol of psychometric tests, including both clinician-rated and self-rated tools, as detailed below. The psychometric assessment was performed by an experienced psychiatrist, trained in the use of the assessment tools described below.

We chose to include several of the available measures for misophonia, as most of them are not fully validated and show intrinsic limitations (for instance, they do not measure the actual magnitude of response to triggers), which is the reason why there have been recent attempts to develop new tools in this field of research (Dibb et al., 2021).

As possible associations have been suggested between misophonia and psychiatric disorders and/or symptoms, including anxiety, depression, and obsessive-compulsive tendencies, in our psychometric assessment, we included an overall measure of psychopathology (Symptom Checklist 90-R) and specific measures for the symptoms detailed above. Some of these measures are clinician-rated to avoid the possible biases of self-rated questionnaires. Last, we included a measure of resilience, which can be defined as the process of adapting well in the face of adversity and stress (American Psychological Association, 2014).

While we clearly expected to find higher scores on the misophonia scales in participants with misophonia when compared with controls, two scenarios could be hypothesized for the psychopathological measures: no difference between participants with misophonia and controls, or higher scores in participants with misophonia compared to controls, which would suggest a possible overlap of the misophonia construct with psychopathological symptoms. Regarding resilience, we were interested in assessing possible differences between the two groups, groups with possibly lower resilience ratings suggesting poorer resources in participants with misophonia in adapting to perceived stressors.

#### Amsterdam Misophonia Scale

This scale is an adaptation of the Yale-Brown Obsessive Compulsive Scale (Y-BOCS) to address misophonic symptoms. It has six items, scored from 0 (no symptom) to 4 (extreme). Scores 0–4 suggest subclinical misophonic symptoms, 5–9 mild, 10–14 moderate, 15–19 severe, and 20–24 extreme (Schröder et al., 2013).

#### Misophonia Questionnaire

The Misophonia Questionnaire (MQ) is a three-part self-rated questionnaire that evaluates the presence of misophonic symptoms, the resulting emotions and behaviors, and the overall severity of sound sensibility. Misophonia symptoms and resulting emotions and behaviors are assessed with 19 items which can be scored from 0 (not at all true) to 4 (always true). Ratings from the first two parts are summed together to produce a total score ranging from 0 to 72. Misophonia sound sensitivity severity is rated on a scale from 1 (minimal) to 15 (very severe) with a score greater than or equal to seven indicating clinical misophonia (McKay et al., 2018). Initial validation of the MQ indicates good internal consistency for the symptom scale (α = 0.86), the emotions and behaviors scale (α = 0.86), and total score (α = 0.89) (Wu et al., 2014).

#### Misophonia Activation Scale

This is a clinician-rated scale focusing on physical and emotional reactions to the trigger sounds in misophonic subjects. Participants are presented with 11 levels of responses to the known and personal misophonic triggers, with higher levels of response reflecting the severity of the disorder. The score ranges from 0 (no reaction to specific sound) to 10 (physical aggression, both self and others-directed) (Fitzmaurice, 2010).

#### Misophonia Assessment Questionnaire

The Misophonia Assessment Questionnaire (MAQ) includes 21 questions about the time spent on thoughts and feelings related to misophonic cues, evaluated on a four-points Likert-type scale ranging from 0 (not at all) to 3 (always true). Scores ranging from 0 to 11 indicate subclinical misophonic symptoms, 12–24: mild, 25–37 moderate, 38–50 severe, and 51–63 extreme (Johnson, 2014).

#### Yale-Brown Obsessive Compulsive Scale

Clinician-rated, 10-item scale, which assesses the severity of obsessions and compulsions in the week prior to the test. Total scores range from 0 to 40 (Goodman et al., 1989).

#### Hamilton Anxiety Rating Scale

This clinician-rated scale consists of 14 items, measuring both the mental anxiety (psychological distress) and somatic anxiety (physical symptoms related to anxiety). The total score ranges from 0 to 56, where <17 indicates mild severity, 18–24 mild to moderate severity, and 25–30 moderate to severe symptoms (Hamilton, 1959).

#### Hamilton Depression Rating Scale

The Hamilton Depression Rating Scale (HAM-D) is a clinician-rated 24-items test. The evaluation for most items is the result of the integration between the objective observation of signs and subjective exposure of symptoms, although the severity criterion mainly refers to the former. Scores 0–9 indicate subclinical depression, 10–13 mild depression, 14–17 moderate depression, and greater than17 indicate severe depression (Hamilton, 1960).

#### State-Trait Anxiety Inventory 1 and 2

It is a 40-item self-administered test for the assessment of state and trait anxiety. Each item is rated from 1 to 4 (1 = not at all, 4 = severe), and no specific cut-offs exist. The higher the score, the higher is the anxiety. Internal consistency coefficients for the scale ranged from 0.86 to 0.95; test–retest reliability coefficients ranged from 0.65 to 0.75 over a 2-month interval. Test–retest coefficients for this measure in the present study ranged from 0.69 to 0.89 (Spielberger, 1989).

#### Resilience Scale for Adults

It is a 33-item self-administered scale evaluating intra- or inter-relational stress-preventing factors (positive self-perception, positive future perception, social competence, structured style, family cohesion, and social resources). The higher the total score, the greater is the subject’s resilience. The Resilience Scale for Adults (RSA) is reliable, with good internal consistency demonstrated by Cronbach alpha values ranging from 0.79 to 0.88 in various studies, while among the six factors, it ranges from 0.67, for the structured style, to 0.81, for the perception of Self (Friborg et al., 2005).

#### Symptom Checklist 90 Revised

The Symptom Checklist 90 Revised (SCL-90-R) is a multidimensional self-report measure that assesses the severity of current psychological symptoms and distress. It assesses nine symptom dimensions: somatization, obsessive–compulsive, interpersonal sensitivity, depression, anxiety, hostility, phobic anxiety, paranoid ideation, and psychoticism. It also includes three global indices of psychological distress: global severity index (number of symptoms endorsed and intensity of distress), positive symptom distress index (average level of distress for those items that were endorsed; exaggerating or attenuating response style), and positive symptoms total (total symptoms endorsed/breadth of distress). All the Italian version subscales show a good internal coherence, with α-values between 0.70 and 0.96 (Derogatis, 1992).

### Analysis of heart rate variability and galvanic skin conductance

In order to achieve our primary endpoint, which was related to the evaluation of the orthosympathetic/parasympathetic balance, we performed analyses of HRV and GSC, which are widely adopted methods for the assessment of the autonomic nervous system (Mackersie and Calderon-Moultrie, 2016). In particular, we expected to find increased parameters of HRV and GSC related to orthosympathetic activation in the participants with misophonia and increased parameters of HRV and GSC related to parasympathetic activation in the HCs.

**FIGURE 2:**
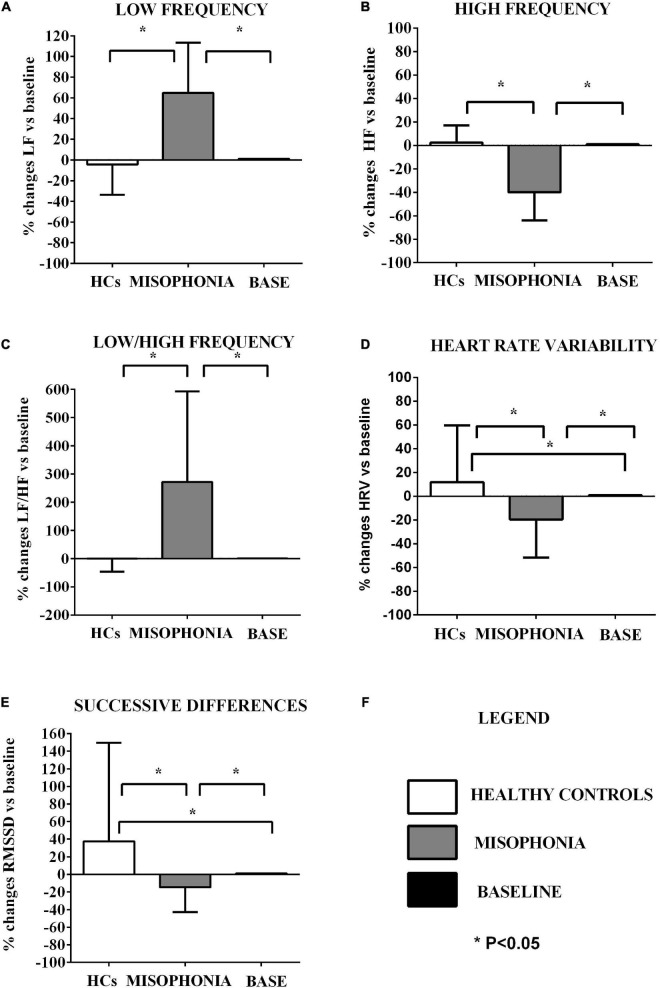
Effects of the trigger sounds protocol on HRV variables in participants with misophonia and participants without misophonia [healthy controls (HCs)] vs. specific baseline (set as 1). The results show increased orthosympathetic variables (LF, LF/HF) in participants with misophonia and increased parasympathetic variables (HRV triangular index, RMSSD) in participants without misophonia in comparison with values registered at baseline. It is also to note that the parasympathetic variables (HF, HRV, and RMSSD) were decreased in participants without misophonia RMS in comparison with values registered at baseline LF, low frequency **(A)**; HF, high frequency **(B)**; LF/HF **(C)**, low frequency/high frequency ratio; HRV **(D)**, heart rate variability triangular index; RMSSD **(E)**, the root mean square of successive differences between normal heart beats. The results are the mean ± SE. The parenthesis indicate significance between groups, as specified in panel **(F)**. In panel **(F)**, the explanation for various groups’ representation is reported. The statistical analysis between M and HCs was performed through the Mann–Whitney test, whereas the statistical analysis between M/HCs and baseline was performed through the Wilcoxon test.

**FIGURE 3:**
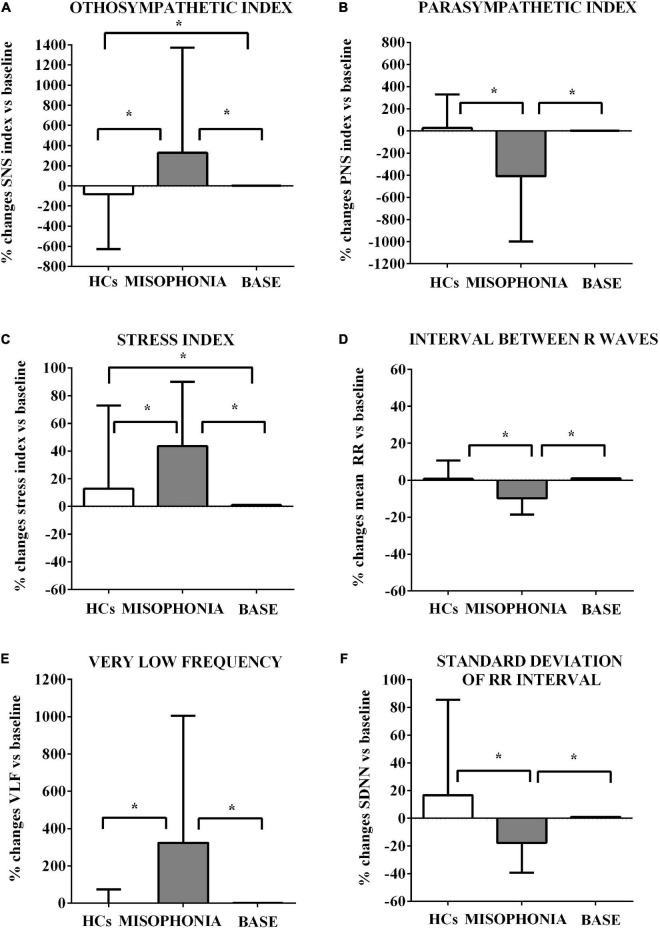
Effects of the trigger sounds protocol on HRV variables in participants with misophonia and participants without misophonia [Healthy controls (HCs)] vs. specific baseline (set as 1). The results show increased orthosympathetic variables (SNS index, Stress index, and VLF) in participants with misophonia in comparison with values registered at the baseline. It is also to note that the parasympathetic variables (PNS index and mean RR) were decreased in participants without misophonia in comparison with values registered at baseline. SNS **(A)**, sympathetic; PNS **(B)**, parasympathetic; stress index **(C)**, stress index; RR **(D)**, interval the elapsed time between ECG R waves; VLF **(E)**, very low frequency; SDNN **(F)**, standard deviation of RR intervals. The results are the mean ± SE. The parentheses indicate significance between groups, as specified in Figure 2F. Various groups are represented as in Figure 2. The statistical analysis between M and HCs was performed through the Mann–Whitney test, whereas the statistical analysis between M/HCs and baseline was performed through the Wilcoxon test.

**FIGURE 4:**
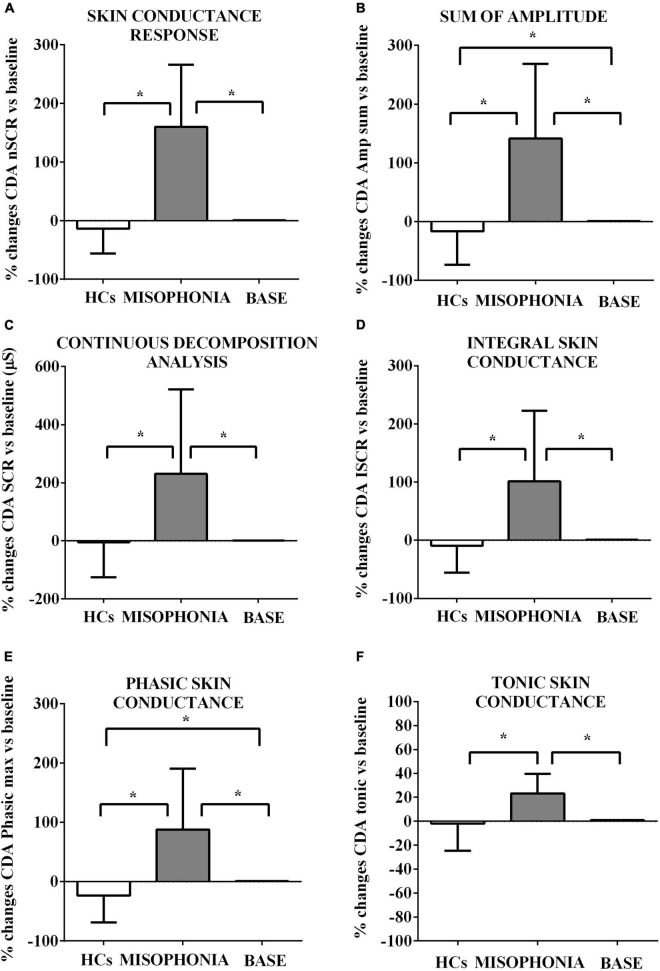
Effects of the trigger sounds protocol on GSC variables in participants with misophonia and participants without misophonia [Healthy controls (HCs)] vs. specific baseline (set as 1). The results show increased orthosympathetic variables (CDA.nSCR, CDA.AmpSum, CDA.SCR, CDA.iSCR, CDA.PhasicMax, and CDA.Tonic) in participants with misophonia in comparison with values registered at the baseline (set as 1). In participants without misophonia, some of those variables related to orthosympathetic activation (CDA.AmpSum and CDA.PhasicMax) were lower in comparison with values registered at the baseline. CDA, continuous decomposition analysis; SCR, skin conductance response; CDA.nSCR **(A)**, SCRs within response window (wrw); CDA.AmpSum **(B)**, sum of SCR-amplitudes of significant SCRs; CDA.SCR **(C)**, continuous decomposition analysis skin conductance response; CDA.iSCR **(D)**, integral of SCR over the 10-s non-overlapping time; CDA.PhasixMax **(E)**, maximum value of phasic activity wrw; CDA.Tonic **(F)**, the mean tonic activity wrw. The results are the mean ± SE. The parentheses indicate significance between groups, as specified in Figure 2F. Various groups are represented as in Figure 2. The statistical analysis between M and HCs was performed through the Mann–Whitney test, whereas the statistical analysis between M/HCs and baseline was performed through the Wilcoxon test.

Heart rate variability and GSC were monitored before (baseline) and during the trigger sounds protocol at the Laboratory of Physiology. In particular, we have extrapolated the numerical values of the entire registrations through special programs, as reported below. Thereafter, values were taken from the baseline and from the end of the trigger sounds protocol and were used to calculate an average value (Figures 2–4) and to make the graphs. All this was done for each sound and for each participant. The HRV was obtained through automatized analysis of electrocardiogram (ECG) by Kubios.

The data from ECG and GSC were recorded together on sitting subjects; furthermore, the subjects were seated in front of a white wall without any possible distraction and were asked to fixate on a point on the wall.

#### Heart rate variability analysis

Heart rate variability parameters were obtained by the computerized analysis of 6-lead ECG performed through Easy ECG pocket and Software Easy View Plus Stress, Ates Medical Device, Colognola Ai Colli, Verona, Italy. Kubios HRV program version 3.1.0 was used to analyze the collected data in both the time domain and frequency domain, as previously described (Tarvainen et al., 2014).

In particular, in the time domain, the following variables have been measured: mean RR (means of RR intervals, at RR intervals, or the elapsed time between ECG R waves), standard deviation of RR intervals (SDNN, for vagal activity) (Gernot, 2017; Forte et al., 2019), and the root mean square of successive differences between normal heart beats (RMSSD, for vagal activity). In the frequency domain, the following variables have been measured: high frequency (HF, for vagal activity), low frequency (LF) (LF, sympathetic activity, or a mix between sympathetic and vagal influences), LF/HF (sympathetic activity), very low frequency (VLF, for sympatho-vagal balance) and the HRV triangular index (for vagal activity).

In addition, sympathetic (SNS), parasympathetic (PNS) index, and the stress index have been examined through Kubios analysis of the registrations.

#### Galvanic skin conductance analysis

In order to collect GSC, a pair of Ag-AgCl electrodes was attached to the palmar surface of the middle and ring fingers of the participant’s non-dominant hand. Prior to attachment, participants’ hands were cleaned with an alcohol wipe and a skin conductance gel was applied to each electrode.

Galvanic skin conductance was recorded with a Shimmer3 GSR+ unit wireless device (Shimmer Research Ltd., Dublin, Ireland), with two 8 mm Ag chloride electrodes (GSR electrodes shimmer sensins), closed by velcro. Between electrodes, a constant potential of 0.5 V was maintained.

The recorder was connected *via* Bluetooth to a portable PC and the recorded data were shown on Consensys Pro (official Shimmer acquisition software) interface PC window, over a range 0–100 micro S (μS). The gain parameter was set at 5 μs/V and A/D resolution was set at 16 bits in order to acquire data over this time range. The sample acquisition rate was 30 samples/s (Gatti et al., 2018).

Analysis of skin conductance data was performed with Matlab version R2015a (The Mathworks, Natick, MA, United States) using Ledalab v3.4.7 and custom analysis programs. The raw skin conductance data were downsampled to 8 Hz and visually examined for the presence of motion artifacts, as indicated by HF fluctuations in the signal amplitude. The relatively few artifacts that were identified were replaced using linear interpolation. The GSC analysis was performed by using a continuous decomposition analysis (CDA) method in order to capture only the variations of the activity, under stimulation, that reflected an effective difference from baseline in each subject (Benedek and Kaernbach, 2010). In this case, period analysis was done only at every 10 s stimulation peak, and the results consisted of the average of GSC during the entire stimulation period.

The CDA was performed to separate the tonic component [skin conductance level (SCL)] and the phasic component [skin conductance response (SCR)] of the signal, using an amplitude criterion of 0.04 μS for defining SCRs, which reveals the activation of the sympathetic nervous system (Benedek and Kaernbach, 2010; Staib et al., 2015; Clark et al., 2018; Posada-Quintero et al., 2020).

Measured variables were as follows: the number of significant (above-threshold) SCRs within the response window (CDA.nSCR), the sum of SCR amplitudes of significant SCRs within the response window (reconvolved from the corresponding phasic driver-peaks) (CDA.AmpSum in μS), the mean tonic activity within the response window (of decomposed tonic component) (CDA.Tonic in μS), the maximum value of phasic activity within the response window (CDA.PhasicMax in μS), and the integral of SCR over the 10-s non-overlapping time (CDA.iSCR in μS × s), which was calculated to represent the overall SCR in a certain time period. The CDA.iSCR is thought to capture the cumulative effect of the signals while avoiding any influences by the usually arbitrary decision of the thresholds for peak detection and event definition (Zhang et al., 2018; Kim et al., 2019).

### Functional magnetic resonance imaging analysis

The objective of the fMRI analysis was to describe the neural correlates of activation in participants with misophonia and to identify any abnormally activated cortical areas compared to healthy subjects. We expected to observe an activation of areas within the central nervous system involved in the control of the orthosympathetic/parasympathetic balance and in the processing of emotions in participants with misophonia during the trigger sounds protocol. Brodmann’s classification system was used for the definition of cortical areas.

#### Resonance magnetic imaging specificity and protocol

A 3-Tesla Magnetic Resonance scanner was used (Ingenia, Philips, Bergen, Norway) with a multichannel head coil (16 channels) and a NordicAktiva (NordicNeuroLab) fMRI system for stimulus administration.

The fMRI protocol was a continuous scanning protocol with two conditions, sound and silence. BOLD sequences, T1-weighted, and T2-weighted sequences were acquired for functional and anatomical reconstructions.

The parameters of T1-weighted sequence were as follows: FOV 130 × 130 × 120, voxel size = 2 × 2 × 4 mm, slice thickness = 4 mm, MPRAGE sequence, TR = 3000 ms, TE = 35 ms, flip angle = 90°, and acquisition matrix = 96 × 94. The parameters of BOLD sequences were as follows: FOV 182 × 240 × 256, voxel size = 1 × 1 × 1, slice thickness = 4 mm, TR = shortest, TE = shortest, flip angle = 8°, and acquisition matrix = 320 × 300.

The sounds were administered according to the trigger sounds protocol; during the fMRI continuous scanning protocol, for each sound, a sequence was repeated, which included four periods of sound presentation (30 s duration each) and 5 periods of silence (30 s duration each) where no sound was emitted, as described above. The total duration of this protocol was 41 min and 18 s (including centering and morphological T1 sequences).

#### Patient preparation

Each participant, before being positioned inside the MRI scanner, was instructed by the radiologist about the execution of the examination, the background sounds, the timing, and the protocol in use. No information was provided on the nature of the sound.

Before starting the acquisition, head coils and soundproofing headphones were used for the subjects (external noise reduction of about 30 dB). The chosen volume amounted to about 70 dB HL, which was set up before the starting fMRI through the machine software. This sound intensity reflects the average intensity value found in everyday life. Therefore, it represents a sound intensity that participants with misophonia may commonly encounter.

At the end of the fMRI scan, oral feedback on the perception of sounds was requested. All participants confirmed that they heard and recognized the sounds.

#### Post-processing analysis

Once the data were acquired, data were converted into DICOM format with the MRIcroGL software and then reconstructed with the SPM12 Software (2020) (SPM12, updated at October 2014). Within- and between-group comparisons were made in results obtained through the fMRI. The statistical performed parameter mapping (SPM) analysis was the same for all subjects and verified by an experienced operator external to the study.

Functional images were realigned to correct for motion, spatially transformed to standard stereotaxic space (based on the Montreal Neurological Institute coordinate system) and smoothed with an 8-mm full-width at half-maximum Gaussian kernel to decrease spatial noise prior to statistical analysis. Rotational motion in degrees (pitch, roll, and yaw), and translational movement in millimeters (x,y,z), were calculated based on the SPM12 parameters for motion correction of the functional images in each subject. No participants had movement greater than 2.5-mm translation or 3 degrees of rotation; therefore, none were excluded from further analysis. One-sample *t*-tests were performed to reveal statistically significant signal differences generated by trigger sounds and the general annoyance sound in participants with misophonia and HCs. The family-wise error rate corrected to *p* < 0.05 was used for all analysis methods (Nandy and Cordes, 2004).

During the fMRI model estimation process, reconstruction was performed for *p*-values = 0.1 (that were not statistically significant) without the family-wise error rate correction, as a control for an actual BOLD signal recording. All participants showed the presence of a signal; therefore, none were excluded from further analysis.

All reported areas having a statistically significant *p*-value and a cluster size greater than 2 were considered as “activated areas” or “activation.” No masks were applied during the post-processing analysis.

### Statistical analyses

The calculation of the sample size was performed based on the primary endpoint, which in our study was defined as an activation of the autonomic nervous system. To do this, we considered a quantitative parameter related to the activity of the orthosympathetic system, that is GSC. Literature data reported a difference in the GSC between HCs and participants with misophonia amounting to 0.15 ± 0.4 μS (Edelstein et al., 2013). Using a statistical power of 80% and an alpha error of 0.05, we obtained a total of 55 subjects divided between 11 participants with misophonia and 44 HCs (controls). Misophonic and control groups were compared through descriptive statistics. Categorical data are reported as a percentage and absolute frequencies. Continuous data are summarized as mean ± standard deviation (SD) or standard error (SE). Wilcoxon-type and Mann–Whitney tests were performed for continuous variables and the Pearson chi-square test or Fisher-exact test, for categorical variables.

A generalized linear mixed effect (GLME) model was used to examine and compare response trends over time (during the trigger sounds protocol vs. baseline), compare group averages (HCs vs. participants with misophonia), and compare the measurement time within each group (interaction term). A random effect (intercept) on the subject’s identification code term has been considered to account for correlations within repeated measurements across time. The time effect with group membership interaction was also considered as a fixed-effect factor to adjust the estimates, together for sex and age. Separate univariable models were estimated. The *p*-values were adjusted for multiple endpoints by considering the Benjamini–Hochberg correction. The model estimates together with the SE and *p*-values are reported. The HRV and GSC results are presented in Figures 2–4 created using GraphPad Prism 6.

*P*-values < 0.05 were considered statistically significant. Statistical analyses were conducted using R 3.5.2 with rms packages (Core and Team, 2019) and lme4 (Bates et al., 2015).

## Results

Data from participants with misophonia and HCs are reported in Table 1. The two groups were significantly different with regard to age, with the misophonic subjects being older than HCs.

**TABLE 1:** Anthropometric variables.

Variables	Participants with misophonia (*n* = 11)	HCs (*n* = 44)	*P*-value
AGE (range)	34.36 ± 12.30 (16–56)	27.55 ± 7.03 (23–56)	0.034
Female %	73% (8)	64% (28)	0.571
Male %	27% (3)	36% (16)	0.571
Weight	69.0 ± 16.3	60.3 ± 10.3	0.073

HCs, healthy controls. Age and weight values are mean ± SD.

### Psychometric assessment

Table 2 shows significant group differences for the psychometric assessment between misophonic subjects and HCs. The most striking result emerging from the psychometric assessment is that, as expected according to the study sampling procedure, subjects in the misophonia group scored significantly higher on all the questionnaires specifically assessing the misophonia construct [Amsterdam Misophonia Scale (A-MISO-S), Misophonia Activation Scale (MAS-1), MQ, and MAQ]. In contrast, we failed to find group differences in the questionnaires assessing the overall psychopathology (Symptom Checklist-90-R), obsessive-compulsive, depressive, and anxiety symptoms [Y-BOCS, HAM-D, Hamilton Anxiety Rating Scale (HAM-A), and State-Trait Anxiety Inventory].

**TABLE 2:** Psychometric assessment.

Test	Participants with misophonia	HCs	*z*-test	*P*-value
A-MISO-S	8.70 ± 6.13	2.50 ± 2.51	−2.58	0.01
MAS-1	4.60 ± 1.84	2.50 ± 1.35	−2.61	0.009
MQ	29.30 ± 15.14	8.70 ± 7.45	−3.09	0.002
MQ sev	5.20 ± 2.90	1.20 ± 1.40	−4.90	0.00
MAQ	21.50 ± 18.32	2.00 ± 3.23	−3.09	0.002
Y BOCS	4.800 ± 6.426	0.100 ± 0.316	−1.69	0.091
HAM-A	9.20 ± 9.68	7.50 ± 5.50	−0.33	0.743
HAM-D	3.00 ± 4.52	2.00 ± 2.45	−0.12	0.907
**STAI**				
STAI S	35.70 ± 11.21	30.40 ± 5.76	−1.55	0.122
STAI T	37.50 ± 7.43	35.10 ± 7.42	−1.26	0.206
**RSA**				
Planned Future	11.20 ± 3.46	14.20 ± 2.15	2.20	0.028
Structured Style	12.40 ± 3.24	17.00 ± 2.98	2.88	0.004
RSA Tot	107.5 ± 21.0	122.5 ± 18.7	1.54	0.123
Self Esteem	20.40 ± 4.38	20.10 ± 4.28	−0.04	0.971
Social Competence	19.90 ± 5.59	19.80 ± 6.09	−0.22	0.827
SCL-90	48.9 ± 42.3	35.5 ± 46.0	−1.03	0.301
Tot	6.20 ± 6.71	5.80 ± 7.54	−0.48	0.634
SCL Som	6.80 ± 6.83	5.00 ± 6.91	−0.74	0.46
SCL Obs-Comp	6.30 ± 5.38	4.80 ± 6.37	−1.00	0.317
SCL Sens	9.70 ± 9.79	5.70 ± 8.64	−0.96	0.335
SCL Dep	4.70 ± 6.04	3.50 ± 4.65	−0.52	0.603
SCL Anx	2.70 ± 2.71	2.10 ± 2.69	−0.48	0.628
SCL Coll-Ost	4.40 ± 4.03	3.30 ± 3.83	−0.78	0.435
SCL Par	2.60 ± 2.07	3.00 ± 5.68	0.87	0.387

HCs, healthy controls. Data are mean ± SD. Mann–Whitney test was used.

Specifically, according to the mean scores on the A-MISO-S and MAQ, participants in the misophonia group had mild levels of misophonia, while those in the HCs scored within normal limits. The mean MQ severity score in the misophonia group, nonetheless, was 5.20, lower than the cutoff (score > 7) indicating possible misophonia.

Another interesting result is the finding of lower resilience scores in misophonic subjects, suggesting that compared to HCs, they possess less resilience when facing stressors.

### Effects of the trigger sounds protocol on heart rate variability and galvanic skin conductance

As reported in section “Materials and methods,” we performed this analysis in order to address our primary endpoint, which was to determine changes in the orthosympathetic/parasympathetic balance in the participants with misophonia vs. participants without misophonia.

First, we measured HRV and GSC variables in both participants with and without misophonia before the start of the trigger sounds protocol in order to collect baseline values of all participants. These data were used for comparison with the results obtained within each group of participants after the trigger sounds protocol. The changes between measurements of HRV and GSC variables obtained during the trigger sounds protocol vs. baseline values (set as 1) were calculated as a percentage. As shown in Table 3, the baseline analysis of HRV and GSC parameters evidenced significant differences between the participants with misophonia and HCs. Hence, all examined variables except the maximum value of phasic activity within the response window, LF, and LF/high frequency were higher in the participants with misophonia than HCs. In addition, high frequency, parasympathetic index, and VLF were lower in the formers. These findings were in agreement with our test hypothesis that the orthosympathetic tone (basal orthosympathetic activity) would be dominant in participants with misophonia, and a parasympathetic tone (basal parasympathetic activity) would be dominant in HCs in the baseline condition.

**TABLE 3:** Baseline HRV and GSC variables.

Variables	Participants with misophonia	HCs	*z*-test	*P*-value
**Heart rate variability time domain**				
RMSSD	43.7 ± 34.3	55.7 ± 29.1	1.71	0.088
MEAN RR	937.3 ± 209.3	980.7 ± 94.9	1.28	0.202
SDNN	47.9 ± 29.4	53.7 ± 22.8	1.07	0.287
HRV	11.43 ± 4.66	10.95 ± 3.67	−0.13	0.901
**Heart rate variability frequency domain**				
LF	39.40 ± 6.49	34.20 ± 4.02	−2.46	0.012
HF	60.59 ± 6.47	65.80 ± 4.02	2.46	0.012
LF/HF	0.6673 ± 0.1773	0.5256 ± 0.0968	−2.46	0.012
VLF	2.84 ± 1.71	3.82 ± 1.02	2.11	0.034
**Kubios parameters**				
SNS Index	0.804 ± 0.603	0.437 ± 0.918	−0.91	0.37
PNS Index	0.451 ± 0.329	1.347 ± 0.983	3.19	0.001
SI	10.61 ± 5.20	9.54 ± 3.31	−0.63	0.533
**Galvanic skin conductance**				
CDA.nSCR	4.340 ± 1.325	3.007 ± 0.660	−3.24	0.001
CDA.AmpSum	0.548 ± 0.231	0.267 ± 0.127	−3.81	<1E-04
CDA.SCR	0.00540 ± 0.00257	0.00363 ± 0.00635	−3.24	0.001
CDA.iSCR	1.821 ± 0.411	1.298 ± 0.377	−3.49	0.0002
CDA.PhasixMax	0.811 ± 0.250	0.857 ± 0.308	0.34	0.744
CDA.Tonic	4.578 ± 1.458	3.413 ± 0.764	−3.11	0.001

The data show increases in the baseline orthosympathetic variables in participants with misophonia (LF, LF/HF, VLF, CDA.nSCR, CDA.AmpSum, CDA.SCR, CDA.iSCR, and CDA.Tonic) and increases in baseline parasympathetic variables (HF, PNS index) in participants without misophonia. LF, low frequency; HF, high frequency; RMSSD, root mean square of successive differences between normal heartbeats; HRV, heart rate variability triangular index; SNS, sympathetic; PNS, parasympathetic; SI, stress index; VLF, very low frequency; SDNN, standard deviation of RR intervals; CDA, continuous decomposition analysis; SCR, skin conductance response; CDA.nSCR, SCRs within response window (wrw); CDA.AmpSum, sum of SCR-amplitudes of significant SCRs; CDA.iSCR, the integral of SCR over the 10-s non-overlapping time windows; CDA.PhasixMax, the maximum value of phasic activity wrw; CDA.Tonic, the mean tonic activity. Values are mean ± SD.

These baseline values were then used to compare the effects of the trigger sounds protocol in the two groups of subjects. It is noteworthy that in comparison with the baseline, in HCs, the trigger sounds protocol caused a significant increase of the HRV triangular index and a decrease of sympathetic index (as% vs. baseline: 11.7 ± 2.7 and −83.2 ± 31, respectively, *p* < 0.05; Figures 2, 3), the sum of SCR-amplitudes of significant SCRs within the response window, and the maximum value of phasic activity within the response window (as% vs. baseline: −16.36 ± 3.2 and −23.7 ± 2.5, respectively, *p* < 0.05; Figure 4). Also, the mean square difference between successive RR intervals showed an increase, which was, however, at the limit of significance (*p* = 0.05; Figure 3).

The analysis of these variables in participants with misophonia showed opposite results (Figures 2, 3). Moreover, the trigger sounds protocol resulted in a strong increase in all GSC parameters in misophonics compared to HCs (as percent vs. baseline, the number of significant, above-threshold, SCRs within the response window: 159.9 ± 12.1; the sum of SCR-amplitudes of significant SCRs within the response window: 141.3 ± 14.5; SCRs within the response window: 230.8 ± 33.1; the integral of SCR over the 10-s non-overlapping time: 101.6 ± 13.8; the maximum value of phasic activity within the response window: 87.8 ± 11.7; the mean tonic activity within the response window: 23.13 ± 1.8 vs. the number of significant, above-threshold, SCRs within the response window: −4.5 ± 6.8; the sum of SCR-amplitudes of significant SCRs within the response window:−16.3 ± 3.2; SCRs within the response window: −4.5 ± 64.9; the integral of SCR over the 10-s non-overlapping time: −9.3 ± 2.6; the maximum value of phasic activity within the response window: −23.7 ± 2.5; the mean tonic activity within the response window: −1.9 ± 1.2; *p* < 0.05; Figure 3), LF, LF/HF, sympathetic index, stress index and VLF (as% vs. baseline, LF: 64.8 ± 5.5; LF/HF: 271.7 ± 36.6; sympathetic index: 327.7 ± 119.2; stress index: 43.5 ± 5.3; VLF: 323.8 ± 77.5 vs. LF: −4.3 ± 1.6; LF/HF: −0.22 ± 2.6; sympathetic index: −83.2 ± 31; stress index: 12.77 ± 3.4; VLF: 0.22 ± 4.1; *p* < 0.05; Figures 2, 3). In addition, a reduction of all other HRV parameters was observed in participants with misophonia vs. HCs (Figures 2, 3).

**FIGURE 5:**
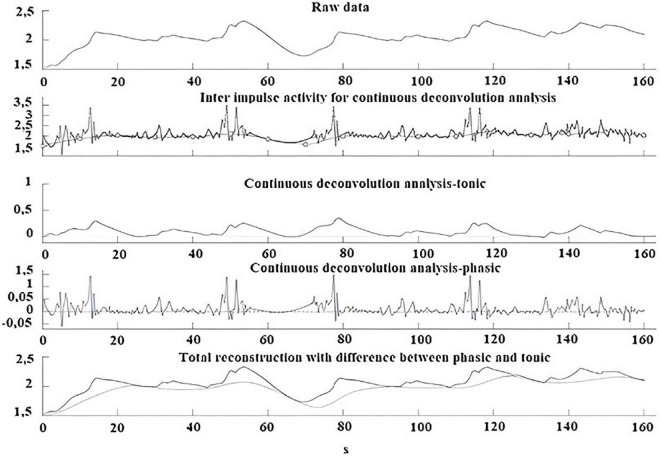
Example of GSC analysis performed during the trigger sounds protocol in a participant with misophonia. From top to bottom: Skin conductance raw data; Inter impulse activity used for continuous deconvolution analysis; Continuous deconvolution analysis-tonic; Continuous deconvolution analysis-phasic; Total reconstruction with differences between phasic and tonic.

An example of GSC analysis taken from one participant with misophonia is shown in Figure 5.

A significant interaction emerged for all parameters indicating that the change in values between the baseline and the trigger sounds protocol showed different patterns for participants with misophonia and HCs. In particular, the values for sympathetic index, stress index, VLF, LF, and LF/HF increased over time among participants with misophonia, whereas, in the same subjects, the values for parasympathetic index, HF, and HRV decreased. When treated as a covariate for modeling group differences, “age” was only a significant factor in two of the N models tested: LF and HF. The GSC values showed increasing trends over time among participants with misophonia (Tables 4, 5).

**TABLE 4:** Linear mixed effect models of the results obtained from HRV.

	Estimate	Standard error	*t*-value	*P*-value	Outcomes
Group^a^	−0.59	0.32	−1.82	0.07	SNS index
Time^b^	1.22	0.13	9.15	<0.001	
Group × Time^c^	−1.97	0.17	−11.91	<0.001	
Age^d^	−0.01	0.02	−0.73	0.46	
Group	0.98	0.36	2.71	0.01	PNS index
Time	−1.57	0.15	−10.67	<0.001	
Group × Time	1.64	0.18	8.99	<0.001	
Age	0.00	0.02	−0.07	0.95	
Group	−1.13	1.47	−0.77	0.44	Stress index
Time	3.42	0.48	7.07	<0.001	
Group × Time	−2.91	0.60	−4.86	<0.001	
Age	0.02	0.07	0.28	0.78	
Group	1.06	0.56	1.87	0.07	VLF
Time^b^	4.04	0.48	8.35	<0.001	
Group × Time	−4.30	0.60	−7.18	<0.001	
Age	−0.01	0.02	−0.40	0.69	
Group	−2.79	1.98	−1.40	0.17	LF
Time	25.22	1.37	18.36	<0.001	
Group × Time	−26.68	1.70	−15.69	<0.001	
Age	0.21	0.09	2.40	0.02	
Group	2.70	1.99	1.35	0.18	HF
Time	−25.08	1.38	−18.23	<0.001	
Group × Time	26.45	1.70	15.53	<0.001	
Age	−0.22	0.09	−2.45	0.02	
Group	−0.05	0.15	−0.35	0.73	LF/HF
Time	1.71	0.12	14.16	<0.001	
Group × Time	−1.72	0.15	−11.47	<0.001	
Age	0.01	0.01	1.59	0.12	
Group	−1.01	1.35	−0.74	0.46	HRV triangular index
Time	−2.75	0.48	−5.72	<0.001	
Group × Time	4.00	0.60	6.71	<0.001	
Age	−0.03	0.06	−0.41	0.68	

All models are adjusted by gender (p > 0.05). ^a^(Group): comparison of values between HCs vs. participants with misophonia. ^b^(Time): comparison of values obtained during the trigger sounds protocol vs. baseline. ^c^Interaction term. ^d^Age as continuous variable.

**TABLE 5:** Linear Mixed effect models about results obtained from GSC.

	Estimate	Standard error	*t*-value	*P*-value	Outcomes
Group^a^	−1.16	0.57	−2.01	0.05	CDA.nSCR
Time^b^	6.92	0.34	20.45	<0.001	
Group × Time^c^	−7.21	0.42	−17.2	<0.001	
Group	−0.35	0.07	−4.64	<0.001	CDA.AmpSum μS
Time	0.68	0.04	16.47	<0.001	
Group × Time	−0.77	0.05	−14.89	<0.001	
Group	0.00	0.00	−0.84	0.40	CDA.SCR μS
Time	0.01	0.00	10.22	<0.001	
Group × Time	−0.01	0.00	−9.40	<0.001	
Group	−0.55	0.22	−2.45	0.02	CDA.ISCR μS × s
Time	1.81	0.14	12.99	<0.001	
Group × Time	−2.05	0.17	−11.86	<0.001	
Group	0.04	0.13	0.28	0.78	CDA.PhasicMax μS
Time	0.81	0.08	10.4	<0.001	
Group × Time	−0.89	0.10	−9.28	<0.001	
Group	−1.08	0.36	−3.00	<0.001	CDA.Tonic μS
Time	1.01	0.08	12.8	<0.001	
Group × Time	−1.15	0.10	−11.72	<0.001	

All models are adjusted by age and gender (p > 0.05). ^a^(Group): comparison of values between HCs vs. participants with misophonia. ^b^(Time): comparison of values obtained during the trigger sounds protocol vs. baseline. ^c^Interaction term.

On the whole, the results obtained provide evidence for a higher orthosympathetic and lower parasympathetic tone at baseline registrations in participants with misophonia than HCs. The state of these tones was then strengthened by the trigger sounds protocol.

### Effects of the trigger sounds protocol on functional magnetic resonance imaging

Functional magnetic resonance imaging was used to characterize the anatomical pathways involved in physiological and emotional responses to the trigger sounds protocol. In particular, it was hypothesized that greater activation in brain regions involving the limbic system, the temporal cortex, and the cerebellum would be observed due to the relationship between these areas and the orthosympathetic/parasympathetic balance. The principal findings are summarized in Table 6.

**TABLE 6:** Functional magnetic resonance imaging (fMRI) activations in participants with misophonia and without misophonia.

Participants with misophonia					Participants without misophonia				
	
Brain area (Brodmann)	Coordinates xyz (mm)			*T*-values	Brain area (Brodmann)	Coordinates xyz (mm)			*T*-values
					
	x	y	z			x	y	z	
41 left	−60	−26	6	6,73	41 left	−60	−28	5	7,3
	−42	−22	4	7,2		−37	−20	4	7,2
	−38	−38	14	6,8		−56	−36	8	6,4
41 right	64	−24	8	6,2	41 right	58	−24	10	7,8
	62	−24	12	6,4		64	−24	12	6,1
	56	−26	10	6,4		43	−24	6	6,5
10 right	22	64	0	7,1					
	44	56	−8						
22 right	60	−24	4	6,1					
	58	−12	−2	7,2					
22 left	−56	−12	2	6,9					
	−68	−36	8	7,3					
Hippocampus	−14	−28	−14	5,5					
Cerebellum sx	−44	−76	−26	6,2					
	−6	−34	−2	5,9					
Cerebellum dx	36	−74	−28	5,4					
	28	−72	−24	6,1					
6 left	−46	−6	54	12,9					
	−54	10	36	9,9					
	−46	−4	56	6,2					
6 right	52	2	48	8,4					
	54	2	48	7,8					
	58	6	16	6,8					
55 right	0	−4	−6	5,3					

Coordinates are expressed according to the Montreal Neurological Institute (MNI) system; brain areas are expressed according to Brodmann’s classification.

The fMRI analysis confirmed our test hypotheses about the involvement of the above neuronal areas in the participants with misophonia during the trigger sounds protocol. Hence, in all cases, a strong activation of the temporal superior gyrus (BA 22; 100%), the temporal cortex (BA 21; 100%), and the auditory cortex (BA 41 and 42; 100%) was found. In about 70% of cases, the premotor cortex (BA 6) and the cerebellum were found to be activated, as well.

Furthermore, 8/11 (73%) participants with misophonia showed activation signals in at least one of the following: the hippocampus, the ventromedial prefrontal cortex, the insula, and the cingulate cortex.

**FIGURE 6:**
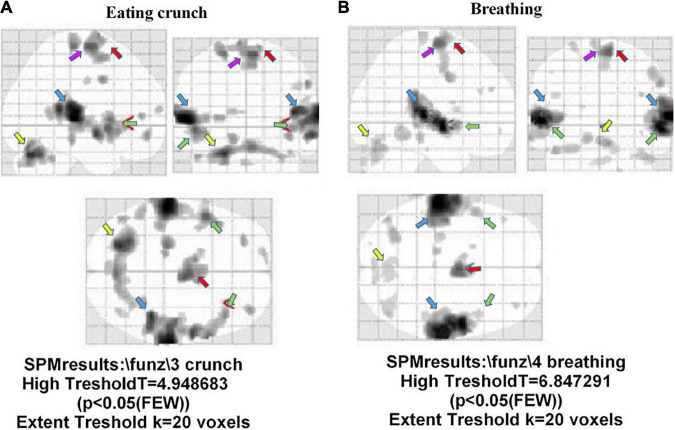
Brain areas activated during the trigger sounds protocol in two participants with misophonia in the three planes. The trigger sounds were “eating crunchy” in panel **(A)**, and “breathing” in panel **(B)**. In panel **(A)**, details on left insula. Also, auditory cortex (BA 41 and 42; light blue arrow), the cerebellum (yellow arrow), the insula (green arrow), the premotor cortex (BA 6; red arrow), and the frontal cortex (BA 4; pink arrow) were activated during the trigger sounds protocol. In panel **(B)**, the areas identified were the auditory areas (BA 41 and 42; light blue arrow), the cerebellum (yellow arrow), and the premotor cortex (BA 6; red arrow).

Figure 6A shows the atypical activation of multiple brain regions in a participant with misophonia after hearing the “eating crunching” sounds. In particular, the auditory cortex (BA 41 and 42), the cerebellum, the insula, the premotor cortex (BA 6), and the frontal cortex (BA 4) were activated during the trigger sounds protocol. Another example of an fMRI image taken in one participant with misophonia is shown in Figure 6B. In that subject, the presentation of “breathing” caused the stimulation of the auditory cortex (BA 41 and 42), the cerebellum, and the premotor areas (BA 6). It is to note that those findings were evidenced after the application of the correction factor “family-wise error rate” in SPM analysis.

Concerning the HCs, 36/44 (82%) subjects demonstrated the activation of the auditory cortex (BA 41 and 42), while in 8 (18%) subjects, we could not find any activated area. In these eight subjects, further post-processing analysis was performed on the images, showing the presence of stimulus response for SPM with *p* = 0.1.

**FIGURE 7:**
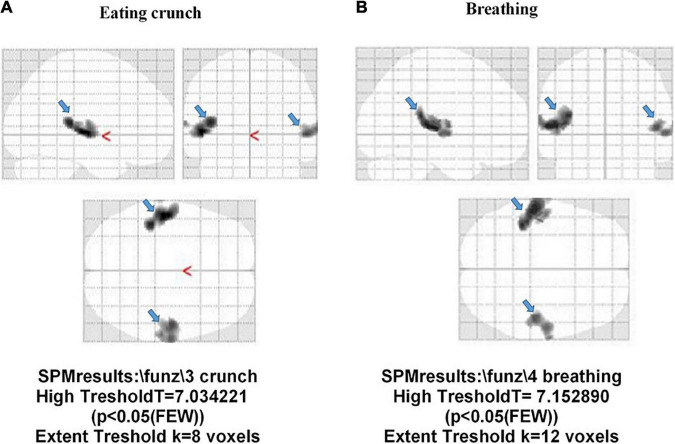
Activation of the auditory cortex (BA 41 and 42; light blue arrow) in two participants without misophonia. The stimulus administered was “eating crunchy” in panel **(A)**, and “breathing” in panel **(B)**.

In Figure 7, examples of fMRI in two HCs are shown; in those subjects, “eating crunching” and “breathing” caused the activation of the primary auditory cortex (BA 42 and 41), only. Also, in this case, as specified above, the correction factor “family-wise error rate” in SPM analysis had been applied.

Thus, the fMRI analysis highlighted the activation of brain regions involved both in the orthosympathetic response and in the processing of trigger stimuli only in participants with misophonia.

## Discussion

The results of this study obtained by combining the fMRI analysis with physiological and psychiatric evaluations showed specific auditory-insula-limbic patterns of activation associated with increased sympathetic tone in participants with misophonia. Those findings are unlikely due to specific psychopathologic features, as shown by the absence of differences between the two groups of participants (those with misophonia and HCs) in the questionnaires assessing obsessive-compulsive, depressive, and anxiety symptoms.

As previously described, misophonia is a disorder characterized by reduced tolerance to specific sounds or stimuli known as “triggers,” which tend to evoke negative emotional, physiological, and behavioral responses (Kumar et al., 2017; Sweedo et al., 2022).

Recently collected data showed a prevalence of participants with misophonia that reaches up to 49% in the general population (Naylor et al., 2021) and about 52% in subjects with obsessive- compulsive disorder (Siepsiak et al., 2020). In addition to feelings of anxiety, distress, and anger, physical manifestations including tightness or pain in the entire body, increased muscular tone, dyspnea, tachycardia, and hypertension have been described (Cavanna and Seri, 2015).

Moreover, those experiences may lead, in some subjects, to a severe decline in daily functioning and the development of behavioral health problems.

Although misophonia now has a consensus definition (Sweedo et al., 2022), and some information is available on the utility of psychometric assessment and involvement of the autonomic nervous system, more in-depth investigations relating to the activation of the orthosympathetic and parasympathetic systems and to the concomitant activation of brain regions involved in emotional responses could be useful for a better understanding of this disorder.

### Heart rate variability and galvanic skin conductance effects induced by the trigger sounds protocol in participants with misophonia

We utilized both HRV and GSC to analyze the autonomic nervous system balance in misophonic participants and HCs (Benedek and Kaernbach, 2010; Tarvainen et al., 2014; Seri, 2015; Staib et al., 2015; Gernot, 2017; Clark et al., 2018; Zhang et al., 2018; Forte et al., 2019; Kim et al., 2019; Posada-Quintero et al., 2020). In particular, we analyzed both the orthosympathetic and parasympathetic drive using HRV and analyzed the orthosympathetic profile using GSC, which is related to the cholinergic-dependent sympathetic stimulation of the cutaneous sweat glands.

The baseline registration of HRV and GSC variables showed interesting results in participants with misophonia. In these participants, HRV variables were more distinctly related to an increased orthosympathetic tone, such as LF and LF/HF and almost all GSC variables. Meanwhile, markers of parasympathetic tone given by HRV, including HF, parasympathetic index, and LF, were higher in HCs than in participants with misophonia. These findings add important information regarding the state of the autonomic nervous system in resting conditions in participants with misophonia vs. HCs and are useful for outlining their orthosympathetic/parasympathetic background.

Participants with misophonia showed increased levels for almost all orthosympathetic HRV measures and decreased levels for almost all parasympathetic measures. The activation of the orthosympathetic system was also confirmed by the GSC analysis. In HCs, it is noteworthy that trigger sounds resulted in a reduction of the sympathetic index, the sum of SCR-amplitudes of significant SCRs, and the maximum value of phasic activity. These results suggest that the trigger sounds protocol caused an inhibition of the orthosympathetic system in HCs unlike what was observed in the participants with misophonia.

Since the main bias of this study was the difference in age between participants with misophonia and HCs, we added a correction for age to the statistical analysis of HRV and GSC data. While we could not confirm our previous observations, since for LF and HF, the statistical significance in results was lost after the correction for age, the general patterns appeared to be similar to those observed without performing the correction for age.

While our findings were similar to those obtained by Kumar et al. (2017), there are several key differences in our experimental protocol that must be considered. For example, Kumar et al. (2017), used two trigger sounds and one neutral sound, whereas we concentrated on six trigger sounds and a general annoyance sound. Furthermore, changes in the autonomic nervous system balance were examined by analyzing many GSC and HRV variables, which evaluated the orthosympathetic/parasympathetic activity both at the baseline and during auditory stimulation. Finally, it should be noted that the sample size in this study was calculated through appropriate statistical tests based on the variation of a parameter related to the activation of the autonomic nervous system (Edelstein et al., 2013). In this way, we could answer the primary hypothesis of the study, which referred to the presence of an altered orthosympathetic/parasympathetic balance in participants with misophonia. In the study by Kumar et al. (2017), instead, participants with misophonia were matched without any power size calculation with participants without misophonia. In addition, in that study, results focused on fMRI analyses. Only one GSC measurement was performed (galvanic skin response), and one HRV measurement (heart rate); no psychometric assessment was provided.

In the study by Edelstein et al. (2013), participants with misophonia were recruited after an interview and the emotional responses to different “trigger sounds” were evaluated. In the second part of that study, a comparison was executed between six participants with misophonia and five HCs in terms of GSC response to auditory–visual stimulations. A similar kind of experimental protocol was executed by Schröder et al. (2019), who conducted psychometric assessments and fMRI analysis in 21 participants with misophonia and 23 HCs. However, a change in the HR was the only measure used to evaluate the autonomic nervous system activation.

As reported above, our findings about the activation of the orthosympathetic nervous system in participants with misophonia are in agreement with previous observations. However, here we conducted a more in-depth evaluation of autonomic nervous system involvement in response to a trigger sounds protocol, by combining it with psychometric and imaging evaluation.

### Neuroanatomical pathways activated by the trigger sounds protocol in participants with misophonia

In participants with misophonia, we observed activations of Brodmann areas 21, 22, 41, and 42, which could be attributed to listening, detection, and understanding of a perceived sound (Pickles, 2015). In addition, in participants with misophonia, the trigger sounds protocol caused activation in the hippocampus, the cingulate cortex, the ventromedial prefrontal cortex, and the insula. Overall, those findings are in agreement with the data by Kumar et al. (2017), who showed that, in participants with misophonia, the trigger sounds were associated with abnormal functional connectivity between the anterior insula cortex, the ventromedial prefrontal cortex, the hippocampus, and the amygdala. It is to note that all of those brain regions are involved with the perception of interoceptive signals and emotion processing (McLachlan, 2009). Moreover, they play a role in the modulation of the autonomic nervous system and in the integration of physiological signals and the dynamic representation of emotional states. In particular, the findings of the involvement of the superior temporal cortex (BA 22), the area with the largest change in the activation in participants with misophonia, could be argued to be related to an increased sensitivity to the trigger sounds in those subjects. In this way, the trigger sounds protocol could have induced auditory attention and caused a greater response to the stimuli, resulting in signals being labeled as emotionally relevant. The absence of activation of the amygdala in participants with misophonia could be attributable to the possible inhibition exerted by the ventromedial prefrontal cortex, which would be involved in the regulation of emotions by the inhibition of neuronal areas involved in this process, such as the amygdala (Motzkin et al., 2015).

Alternatively, it is possible that we did not find any involvement of amygdala because this region is more associated with fear than representing a primary emotional site, as reported by Schröder et al. (2019).

The HCs group showed the activation of Brodmann areas 41 and 42, only, with a mean cluster size of 130 voxel. This could be explained by the trigger sounds protocol that, when compared to the background noise of the fMRI continuous protocol, was of low intensity. Moreover, in eight HCs, there was no activation. Those eight HCs stated during the brief interview following the fMRI that they perceived and recognized the sounds during the examination. These were subjects whose fMRI images were acquired on different days and did not belong to a single session. In order to exclude mistakes in subject sampling, reconstruction analyses were followed for SPM values with *p*-values above the significant threshold (*p* = 0.1). It is to note that this post-processing reconstruction showed activations of Brodmann areas 41 and 42. This activation, in eight HCs, also persisted for values of *p* = 0.05. However, the application of the family-wise error rate resulted in its elimination.

### Psychiatric assessment: Comparison of participants with misophonia and healthy controls

The results of the psychiatric evaluation indicate that misophonia could be a construct independent of the general psychopathology (as assessed with the Symptom Checklist-90 R), anxiety (HAM-A, State-Trait Anxiety Inventory), depression (HAM-D), and obsessive-compulsive features (Y-BOCS).

The currently available literature has described possible associations of misophonia with a variety of psychiatric symptoms, such as traits of obsessive-compulsive personality disorder, mood disorders, attention-deficit (hyperactivity) disorder, autism spectrum conditions (Jager et al., 2020a), post-traumatic stress disorder, and anorexia (Erfanian et al., 2019). Nonetheless, data about the frequency of misophonia in patients suffering from psychiatric disorders are lacking (Siepsiak et al., 2020). Recent research supported the possible relationship of misophonia symptoms with clinician-rated symptoms of personality disorders, but not other psychiatric disorders, even though anxiety was found to partially mediate this relationship (Cassiello-Robbins et al., 2021).

There are also suggestions that misophonia could be a discrete psychiatric disorder, with corresponding implications for treatment (Schröder et al., 2013). Features, such as neuroticism, impulsivity, and difficulties with emotion regulation have been suggested as important risk factors and treatment targets for adults with misophonia (Cassiello-Robbins et al., 2020). Recently, both short-term and long-term efficacy of CBT for misophonia have been reported (Jager et al., 2020b).

In support of our baseline hypothesis, we found higher scores on the misophonia scales in participants with misophonia when compared to HCs. Nonetheless, it has to be underscored that participants in the misophonia group did not score above the cutoff established for misophonia in all scales; actually, they had mild misophonia according to the mean scores on the A-MISO-S and MAQ, but scored lower than the cutoff on the MQ. While this might lead one to argue whether the differences we found between the two groups could be clearly attributed to misophonia (Schröder et al., 2017), we also have to underscore that, as described in the “Materials and methods” section, our assessment included more than one questionnaire, the involvement of a psychiatrist, and proper screening in a face-to-face interview where information about co-morbidity was gathered.

As far as psychopathology is concerned, our first hypothesis is supported, as our results did not evidence the presence of psychopathologic symptoms in participants with misophonia (as assessed with the Symptom Checklist-90 R, HAM-A, State-Trait Anxiety Inventory, HAM-D, and Y-BOCS). Thus, the higher scores found in the misophonia measures seem independent of the overall psychopathology, leading to the possibility that misophonia may be either a discrete pathological condition or a non-pathological variant of human nature linked to a particular way of processing trigger sounds.

Also, misophonia could be involved in the creation of the so-called “global emotional moment.” This is meant as the set of homeostatic, environmental, hedonic, motivational, social, and cognitive activities (Bud, 2009), which contribute to one’s feelings and represent the sentient self at one moment in time.

Finally, the finding of lower resilience scores in participants with misophonia may suggest poorer resources in this group of subjects when facing stressors.

### Connections between neuroanatomic pathways, autonomic nervous system response, and psychiatric assessment in participants with misophonia

The primary auditory cortex lies in the transverse temporal gyri of Heschl that are juxtaposed to the insula, which is considered a key brain area in the homeostasis of visceral information processing and interoception. In particular, the insula is involved in the control of the autonomic nervous system and in the integration of physiological signals, as well as the dynamic representation of emotional states to create the “global emotional moment” (McGeoch and Rouw, 2020). Negative emotional experiences have been reported to induce anterior insular activation, including disgusting, frightening, happy, sad, or sexual images (Uddin et al., 2017).

The cingulate cortex, which is connected to the amygdala, is responsible for the processing of emotions and for the regulation of associated endocrine and autonomic responses. This region is also involved in reward-related processing of endocrine and autonomic responses to them due to the fact that this area is involved in reward-related processing. The cingulate cortex can be considered a connecting center of emotions, sensations, and action. Due to its links with the hippocampus and amygdala, the cingulate cortex could also have a role in the consolidation of long-term memories and the processing of emotionally relevant stimuli. The integration of signals originating from all the above areas would, thus, result in the modulation of the autonomic nervous system drive and in processing emotional states. In predisposed subjects, an auditory-insular synesthesia model could account for the onset of psychiatric symptoms and clinical manifestation of changes in the orthosympathetic/parasympathetic balance. In this way, a dynamic process of altered neurological activation could turn the specific auditory stimuli into trigger sounds to induce strong emotional responses (Nagai et al., 2007; Rolls, 2019). In this context, our data would also support the concept of wellbeing that has been linked to the balance between the two sides of the autonomic nervous system. In particular, it has been hypothesized that conditions of chronic sympathetic hyperactivity and parasympathetic hypoactivity would be associated with reduced emotional wellbeing and a variety of mental disorders and *vice versa* (Thayer and Brosschot, 2005; Strigo and Craig, 2016).

The findings of increased baseline sympathetic variables in participants with misophonia and parasympathetic variables in HCs measured with HRV and GSC would confirm the above issues regarding the reduced “emotional wellbeing reserve” and increased predisposition to undergo changes in wellbeing in response to stressful auditory conditions.

With regard to resilience, which was found to be reduced in participants with misophonia, and in general emotional states, numerous studies have associated this trait with the activation of the mesocorticolimbic area, such as the hippocampus and the prefrontal cortex (Richter et al., 2019). Similarly, Tan et al. (2018) correlated heartbeat interoception and anxious state and found the activation of the insula to be related to this emotional state. Cholinergic signaling in the hippocampus has also been described to regulate the social stress resilience and anxiety- and depression-like behavior. Finally, this study found the activation of the cingulate cortex activation in misophonia, which would be involved with homeostatic motivations that guide an adaptive behavior. Taken together, the insula and limbic areas could represent the neuroanatomical basis linking orthosympathetic system activation, emotion, and feelings (Strigo and Craig, 2016).

A novel finding from this study involved motor control, such as Brodmann area 6 and the cerebellum. The premotor cortex (BA 6), in particular, would be activated “during motor imagery,” that is, when the subject visually imagines a movement or imagines the sensations, he would experience during that movement. Thus, it could be hypothesized that in participants with misophonia, the trigger sounds protocol would activate the neural pathways implicated in the preparation/execution of escape (Kumar et al., 2021).

## Conclusion

In conclusion, the results obtained from our study underline the existence of a specific response pattern within the auditory cortex-insula-limbic areas which, in the presence of “trigger sounds,” would be activated in predisposed subjects, such as participants with misophonia. The recruitment of those neuronal patterns would, in turn, alter the autonomic nervous system balance in favor of the orthosympathetic drive, influencing the emotional wellbeing.

Considering the limitations of the study, future work should investigate different activation patterns resulting from various trigger sounds and the general annoyance sound. Multimodal stimulation using video clips and sounds that simulate everyday life may also reveal patterns similar to those described in this study. Moreover, future comparisons should include a “no annoyance control condition.” The selection of participants should also be based on the recent consensus definition of misophonia (Sweedo et al., 2022), which was not available at the time of this study, and the enlarged sample size to determine specific effects of gender. Finally, any bias related to differences in age could be avoided by specific selection of participants, which would allow for an age correction (this was not performed in the current study).

## Data availability statement

The original contributions presented in this study are included in the article/supplementary material, further inquiries can be directed to the corresponding author.

## Ethics statement

The study was approved by the local Ethical Committee, Azienda Ospedaliero-Universitaria Maggiore della Carità, Novara (CE 81/18). Written informed consent to participate in this study was provided by the participants’ legal guardian/next of kin.

## Author contributions

EG, AS, CG, AC, and PZ: conception of the work. DD, RC, BG, DN, and AF: data collection. AS, DD, BG, DN, DA, DF, and AC: data analysis and interpretation. EG, AS, CG, RC, DF, AC, and PZ: drafting the manuscript. All authors critically revised the manuscript, contributed to the article, and approved the submitted version.
